# Use of the Salivary Secretion

**Published:** 1847-10

**Authors:** 


*Use of the Salivary Secretion.* By M. Bernard.—The recent researches of Mialhe and others tended to show that saliva contains a ferment capable of changing starch into sugar. The experiments upon which was founded this opinion consisted in chemical researches on the fluid escaping, during a given time, from the mouths of animals. M. Bernard derives from a new series of experiments a contrary opinion. Instead of merely collecting the buccal secretions, he took the saliva from the glands themselves, and states that in this unmixed and pure condition that fluid is incapable of causing saccharine fermentation in starch. Pursuing his experiments, M. Bernard separated from the mouth of a dead horse several shreds of the mucous membrane, and found that after prolonged desiccation, they still retained the power of transforming starch into sugar. It is, therefore, the mucous membrane, and not the saliva itself, which causes this change in amylaceous substances. The function of saliva is then simply, according to M. Bernard, to moisten the alimentary bolus, and to connect its various parts into a homogeneous paste. *Ibid.*

